# Effects of Carbohydrates on Rosmarinic Acid Production and In Vitro Antimicrobial Activities in Hairy Root Cultures of *Agastache rugosa*

**DOI:** 10.3390/plants12040797

**Published:** 2023-02-10

**Authors:** Hyeon Ji Yeo, Min Jae Kwon, Sang Yeon Han, Jae Cheol Jeong, Cha Young Kim, Sang Un Park, Chang Ha Park

**Affiliations:** 1 Biological Resource Center, Korea Research Institute of Bioscience and Biotechnology (KRIBB), 181 Ipsin-gil, Jeongeup 56212, Republic of Korea; 2 Department of Biological Sciences, Keimyung University, 1095 Dalgubeol-daero, Dalseo-gu, Daegu 42601, Republic of Korea; 3 Department of Crop Science, Chungnam National University, 99 Daehak-ro, Yuseong-gu, Daejeon 34134, Republic of Korea; 4 Department of Smart Agriculture Systems, Chungnam National University, 99 Daehak-ro, Yuseong-gu, Daejeon 34134, Republic of Korea

**Keywords:** *Agastache rugosa*, hairy root cultures, rosmarinic acid, antibacterial effect

## Abstract

*Agastache rugosa* (popularly known as Korean mint) belongs to the Lamiaceae family and comprises 22 species of perennial aromatic medicinal species native to East Asian countries, such as Korea, Taiwan, Japan, and China. *A. rugosa* contains many phenolic compounds that exhibit pharmacological and physiological activities, including antioxidant, anticancer, antiviral, antifungal, and antibacterial activities. The highest concentrations of rosmarinic acid and its isomers have been reported in the roots of *A. rugosa*. In this in vitro study, hairy roots of *A. rugosa* were obtained and the carbohydrates (sorbitol, mannitol, glucose, maltose, galactose, mannose, and sucrose) were evaluated to determine those that were optimal for rosmarinic acid production and hairy root growth. Antioxidant and antibacterial activities of extracts of *A. rugosa* were also assessed. The best carbon source for *A*. *rugosa* hairy root cultures was sucrose, considering biomass productivity (0.460 ± 0.034 mg/30 mL), rosmarinic acid production (7.656 ± 0.407 mg/g dry weight), and total phenolic content (12.714 ± 0.202 mg/g gallic acid equivalent). Antioxidant and antimicrobial activities were displayed by *A*. *rugosa* hairy roots cultured in liquid medium supplemented with 100 mM sucrose. Twenty-five bacterial strains, including multidrug-resistant bacteria and one pathogenic yeast strain, were used for antimicrobial screening of *A*. *rugosa* hairy roots. The hairy root extracts displayed antibacterial activity against *Micrococcus luteus* (KCTC 3063) and *Bacillus cereus* (KCTC 3624). The inhibition of these bacteria was greater using *A*. *rugosa* hairy roots with the highest levels of phenolic compounds cultured in the presence of sucrose, compared to hairy roots with the lowest levels of phenolic compounds cultured in the presence of fructose. Considering hairy root biomass, phenolic compound production, and antibacterial activity, sucrose is the best carbon source for *A*. *rugosa* hairy root cultures.

## 1. Introduction

*Agastache rugosa*, known as the Korean mint, belongs to the Lamiaceae family found in East Asia (China, Korea, and Japan). It is a traditional medicinal and ornamental plant [1]. In particular, *A. rugosa* has been a traditional herbal remedy for the treatment of miasma, cholera [2,3,4,5], anorexia, and vomiting [6]. *A. rugosa* can be used for physiological and pharmacological treatments, including antifungal [7], antibacterial [8], anticancer [9], and antiviral activities [10]. Furthermore, Park et al. [11] reported that different tissues (flower, stem, and leaf) contained phenolic compounds (catechin, chlorogenic acid, caffeic acid, ferulic acid, rutin, and kaempferol), and that methanolic extracts of the different plant tissues displayed antibacterial activity. Desta et al. [12] described the antioxidant activities of extracts of flower, root, stem, and leaf tissues of *A. rugosa*, which were rich in phenolic compounds. In particular, this study reported that *A. rugosa* roots contain high amounts of rosmarinic acid and its isomers compared with flower, leaf, and stem tissues [12].

Rosmarinic acid is a naturally occurring phenolic carboxylic acid that is commonly found in the *Lamiaceae* family. The family includes herbal plants such as *Rosmarinus officinalis* (rosemary), *A*. *rugosa* (Korean mint), *Salvia officinalis* (sage), *Ocimum tenuiflorum* (basil), *Origanum vulgare* (oregano), *O. majorana* (marjoram), and *Melissa officinalis* (lemon balm) [13]. Rosmarinic acid biosynthesis begins with formation of the 4-coumaroyl-4′-hydroxyphenyllactic acid precursor, which can be biosynthesized from two amino acids (phenylalanine and tyrosine). The precursor is subsequently converted to caffeoyl-4′-hydroxy phenyllactic acid, followed by conversion to rosmarinic acid [14]. Biological properties of rosmarinic acid include antioxidant [15] and antibacterial activities [16].

*Agrobacterium rhizogenes*-mediated transformation of a variety of hairy roots from many plant species has resulted in genetic stability, rapid root growth, and biosynthesis of bioactive compounds. Indeed, hairy roots have been obtained from many plant sources for the production of most groups of secondary metabolites, including phenolics, alkaloids, glucosinolates, terpenoids, and others. Many previous studies have confirmed that hairy roots can be a good source of plant-derived bioactive compounds, with flavonoid (anthocyanin) accumulation in hairy roots of *Fagopyrum tataricum* [17], flavone accumulation in hairy roots of *Scultellaria baicalensis* [18], ginsenoside production in hairy roots of *Platycodon grandiflorum* [19], glucosinolate production in hairy roots of *Nasturtium officinale* [20], resveratrol in hairy roots of *Arachis hypogaea* [21], tropane alkaloids in hairy roots of *Hyoscyamus reticulatus* [22], and tanshinone in hairy roots of *Salvia miltiorrhiza* [23].

A supply of carbohydrates is necessary for root cultures; these sugars are used as energy sources to survive in vitro and contribute to root growth and regeneration. Furthermore, the type of carbohydrate (monosaccharides and disaccharides) reportedly influences primary and secondary metabolism in root cultures. Park et al. [24] reported the enhanced biomass productivity and production of flavones (baicalin, baicalein, and wogonin) according to the sugar types (monosaccharides and disaccharides). Vinterhalter et al. [25] demonstrated optimized root growth and phenolic and xanthone production in *Gentiana dinarica* hairy roots using sucrose, fructose, and glucose, respectively, as the carbohydrate source at different concentrations. Additionally, the accumulation of withanolide A was reported to depend on the carbon source (sucrose, glucose, fructose, maltose, or a combination of sugars) in hairy roots of *Withania somnifera* [26].

Desta et al. [12] described the presence of high levels of phenolic compounds in leaves, flowers, stems, and roots of *A. rugosa*. Roots contained higher concentrations of rosmarinic acid and its isomers compared with the levels in the other tissues [12]. Several studies have reported that a supply of carbon sources is required for hairy root cultures of many plant species [24,25,26]. However, there are little studies on the effect of different carbohydrates on the growth and resveratrol production in hairy roots of *A. rugosa*. In the present study, *A. rugosa* hairy root induction was performed to evaluate the potential of sustainable medicinal bioresources for the production of rosmarinic acid. The optimal conditions for sustainable hairy root growth and rosmarinic acid production were evaluated using eight different carbon sources during culture. Additionally, the antioxidant and antibacterial properties of hairy root extracts were measured to gauge the potential therapeutic value of *A*. *rugosa* hairy roots.

## 2. Results

### 2.1. Establishment of A. rugosa Hairy Root Cultures

**Figure 1:**
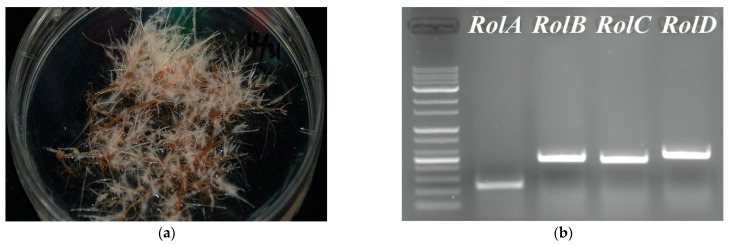
(**a**) *A*. *rhizogenes*-mediated hairy root culture in *A*. *rugosa*; (**b**) PCR analysis of rol A (233 bp), B (483 bp), C (464 bp), and D (525 bp) genes in hairy roots of *A*. *rugosa*.

*A. rhizogenes* harbors a root-inducing plasmid (Ri-plasmid) with *rol* genes A, B, C, and D as transfer-DNA (T-DNA). Infection of host plants with *A. rhizogenes* introduced the T-DNA into the genome of the host plant, facilitating the induction of hairy roots from the plants. T-DNA introduction and transformation were confirmed by PCR analysis of *rol* genes, and transformation can be judged. The PCR analysis of *rol* genes and subsequent gel electrophoresis confirmed that *A. rugosa* hairy roots exhibited bands for the expected PCR product size, including *rol* A (233 bp), B (483 bp), C (464 bp), and D (525 bp) (Figure 1). The findings indicated that the induced roots were hairy roots originating from *A. rugosa* explants.

### 2.2. Growth Patterns of A. rugosa Hairy Root Cultures

*A. rugosa* hairy roots were incubated in half-strength MS broth with 100 mM of the selected carbohydrate (mannitol, sorbitol, glucose, fructose, galactose, mannose, maltose, or sucrose). The dry weight in *A. rugosa* hairy roots was evaluated after harvesting three biological replicates (Table 1). Maximum growth was obtained in the presence of galactose (0.462 ± 0.028 g dry weight (DW)/30 mL) and sucrose (0.460 ± 0.034 g DW/30 mL). The least growth occurred in the presence of mannitol (0.196 ± 0.015 g DW/30 mL), sorbitol (0.189 ± 0.023 g DW/30 mL), and mannose (0.193 ± 0.016 g DW/30 mL). These findings suggest that mannitol and sorbitol sugar alcohols are not suitable for the growth of *A. rugosa* hairy roots, while galactose, glucose, and sucrose are appropriate sugar sources for hairy root growth.

### 2.3. Rosmarinic Acid Production and Total Phenolic Contents Using Eight Different Sugar Sources

Table 2 shows the patterns of rosmarinic acid production by hairy roots in half-strength MS medium supplemented with 100 mM mannitol, sorbitol, glucose, fructose, galactose, mannose, maltose, and sucrose. Rosmarinic acid values ranged from 2.069 ± 0.081 (fructose) to 7.656 ± 0.407 mg/g DW (sucrose), a difference of 3.70 times. The highest value for sucrose was followed in order by high levels for galactose, mannose, and glucose; medium levels for maltose, mannitol, and sorbitol; and the low level for fructose. The addition of sucrose resulted in the highest level of rosmarinic acid which was 3.70 times higher than that of fructose, the lowest value. Additionally, the high levels of rosmarinic acid production were observed in hairy roots grown in galactose (6.061 ± 0.359 mg/g DW), mannose (5.448 ± 0.810 mg/g DW), and glucose (5.538 ± 1.234 mg/g DW), as well and medium the middle levels of rosmarinic acid accumulation were observed in hairy roots grown in maltose (4.682 ± 1.144 mg/g DW), mannitol (4.551 ± 0.529 mg/g DW), and sorbitol (4.474 ± 0.396 mg/g DW).The findings indicate that *A*. *rugosa* hairy root, as a sustainable bioresource, can be used for rosmarinic acid production.

The total phenolic content of hairy roots cultured in the different carbon sources, expressed as mg/g gallic acid equivalent (GAE), decreased in the order of sucrose, fructose, mannitol, sorbitol, glucose, maltose, galactose, and mannose (Table 3). These findings are similar to those of rosmarinic acid. The highest level of total phenolics was recorded in hairy roots cultured in sucrose, followed by galactose, glucose, mannitol, mannose, maltose, sorbitol, and fructose. The findings in Table 2 and Table 3 demonstrate that, of the tested carbohydrates, sucrose is the best for production of rosmarinic acid and total phenolics.

### 2.4. In Vitro Antibacterial Properties of A. rugosa Hairy Root Cultures

**Figure 2:**
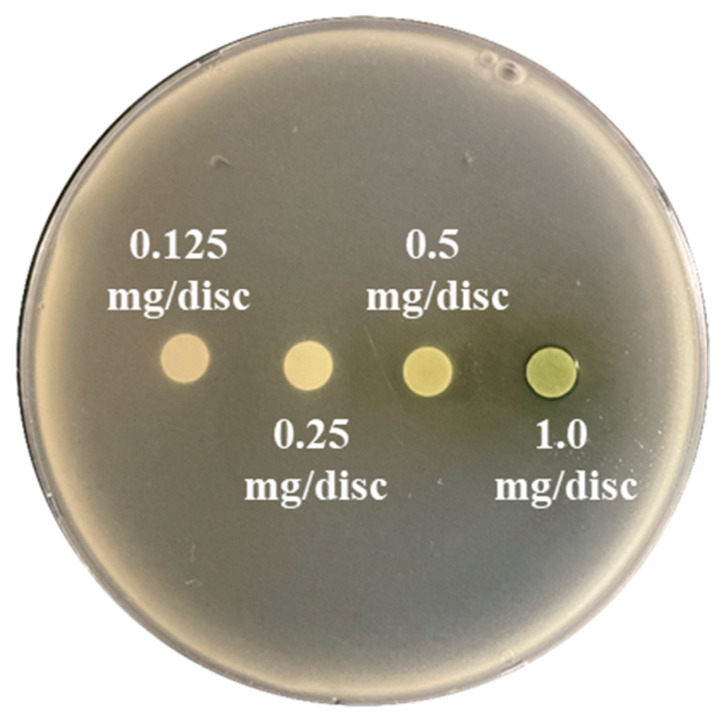
Representative image showing antibacterial activity of *A*. *rugosa* hairy roots cultured with sucrose against *B*. *cereus*.

Zones of growth inhibition surrounding the paper discs containing *A*. *rugosa* hairy root extracts cultured with different concentrations of sucrose (Figure 2 displays representative results) or fructose were determined to assess in vitro antimicrobial activities (Table 4). Growth was inhibited by the methanol extracts from *A*. *rugosa* hairy roots on the agar plates containing *M*. *luteus* (KCTC 3063) and *B*. *cereus* (KCTC 3624). Growth was not inhibited on the plates containing the select multidrug-resistant bacteria, bacterial pathogens, and one opportunistic yeast (Table 4).

## 3. Discussion

*A*. *rugosa* hairy roots were successfully generated from in vitro cultured plants by infection with *A*. *rhizogenes* strain R1000 (wild type), as confirmed by PCR analysis of *rol* genes A–D (Figure 1). The hairy roots were able to produce rosmarinic acid; concentrations ranged from 2.069 ± 0.081 to 7.656 ± 0.407 mg/g DW according to the carbon source. Similarly, Kim, Oh, and Lee [27] reported that all hairy roots generated by five different strains (13333, 15834, R1000, R1200, and R1601) of *A*. *rhizogenes* produced rosmarinic acid, ranged from 14.3 ± 1.3 to 22.6 ± 2.1 mg/g DW, with the highest levels produced by strains R1000 and R1601. Furthermore, Lee et al., 2008 reported that the rosmarinic acid content of *A*. *rugosa* hairy roots reached its maximum after 14 days of culture with 116 mg/g DW [4]. This might be due to different root culture conditions, such as gaseous atmosphere for oxygen supply and carbon dioxide exchange, nutrients, minerals, temperature, and pH [28]. Additionally, previous studies reported that electrical conductivity [29], root zone temperature [30], and indole-3-acetic acid (IAA) [31] treatment affected the accumulation of rosmarinic acid in the intact plants of *A*. *rugosa* and its roots contained higher levels of rosmarinic acid compared with those of the other organs. In particular, the intact roots produced rosmarinic acid contents ranging from 10.163 to 21.141 mg/g DW according to the electrical conductivity conditions [29], ranging from 21.083 to 37.915 mg/g DW according to the root zone temperature [30], and ranging from 10.348 to 29.925 mg/g DW according to the IAA concentrations [31], respectively. Considering these studies, hairy roots of *A*. *rugosa* can be a good source for the production of resveratrol due to its high levels of rosmarinic acid comparable to those of roots of intact plants.

The supply of carbohydrates as an energy source is necessary for in vitro root cultures, including seedling, adventitious, and hairy roots. Among them, sucrose has been used for plant cell and tissue cultures because most plant species contain this disaccharide in their phloem sap and because sucrose is readily available and cost-effective [24,32,33,34]. In this study, supplementation with eight carbohydrates affected root growth and rosmarinic acid production in *A*. *rugosa* hairy roots. Sucrose contributed to the highest productivity of hairy root biomass and rosmarinic acid accumulation. These findings are consistent with prior observations of enhanced natural product accumulation in root cultures supplemented with different carbohydrates. Previously, sucrose was the best source for root growth and production of baicalin, baicalein, and wogonin in *Scutellaria baicalensis* hairy roots [24], and for biomass productivity and accumulation of betacyanin and betaxanthin in *Beta vulgaris* hairy roots [35]. Praveena and Murthy [26] reported that the supply of sucrose resulted in higher root biomass and production of withanolide A in *Withania somnifera* hairy roots compared with the other carbohydrate sources. Verma et al. [36] reported that *Picrorhiza kurroa* hairy root cultured with sucrose displayed higher growth rate and productions of kutkoside and picroside I than the other nine carbohydrate sources examined. Furthermore, galactose showed the highest productivity of hairy root biomass comparable with that of sucrose and the second highest production of rosmarinic acid and total phenolics in *A*. *rugosa* hairy roots. Similarly, Park et al. [24] reported that galactose at a concentration of 100 mM was most beneficial to root growth and production of baicalin, baicalein, and wogonin in *S*. *baicalensis* hairy roots, and Guo et al. [37] reported that supplementation with galactose yielded the highest growth values and production of rosmarinic acid in *Salvia castanea* hairy roots. Thus, this study also suggested that galactose can be a good carbon source for the production of rosmarinic acid in *A*. *rugosa* hairy roots.

In the present study, methanol extracts from *A*. *rugosa* hairy roots cultured with sucrose and fructose, respectively, were used for antimicrobial screening. The extracts displayed antibacterial activities against two pathogens (*M. luteus* and *B. cereus*). *M*. *luteus* causes infections that include pneumonia, bacteremia, endocarditis, and peritonitis in immunosuppressed patients [38]. *B*. *cereus* is responsible for emetic and diarrheal syndrome food poisoning [39]. These extracts did not display antimicrobial activities against multidrug-resistant bacteria and a pathogenic yeast. Similar to these results, essential oils from *A*. *rugosa* leaves reportedly had antibacterial effects against *B*. *cereus* [8]. However, until the current study, the antimicrobial properties of extracts of *A*. *rugosa* hairy roots against *B*. *cereus* and *M*. *luteus* have been unclear. Additionally, antibacterial activities were higher in hairy roots cultured with sucrose than in those cultured with fructose. This might be due to the higher levels of rosmarinic acid and total phenolics in hairy roots cultured with sucrose. Previous studies have reported that rosmarinic acid possesses antibacterial properties against *B*. *cereus* and *M*. *luteus* [40,41,42] and antimicrobial activity of plant phenolic compounds [43]. In this study, the enhanced production of rosmarinic acid and total phenolic compounds in *A*. *rugosa* hairy roots cultured with sucrose and greater antibacterial activity of the extracts are supported by previous studies reporting that *A*. *rugosa* flowers contain higher levels of phenolic compounds and have more pronounced antibacterial properties than those of leaves and stems [11]. White flowers of *Angelica gigas*, containing higher levels of phenolic compounds, showed higher antibacterial activities than those of pink and violet flowers [44]. However, we observed that the growth of only two bacterial pathogens (*M*. *luteus* and *B*. *cereus*) was inhibited by *A. rugosa* hairy root extract. This might be because only 1 mg of extract was loaded onto the discs. In further studies, antibacterial properties against the pathogens tested in this study should be confirmed using higher concentrations of *A. rugosa* hairy root extract. Furthermore, essential oils derived from plants (arborvitae, cassia, clove, lemongrass, oregano, tea tree, and thyme) displayed antimicrobial activities against multidrug-resistant pathogens (*Enterobacter cloaceae*, *Proteus mirabilis*, *Morganella morganii*, *P*. *aeruginosa*, and *E*. *coli*) [45]. Thus, antibacterial activities against multidrug-resistant pathogens should be performed with essential oils derived from *A*. *rugosa* in further studies.

## 4. Materials and Methods

### 4.1. Hairy Root Induction and Cultures

Hairy root induction began with the infection of *A*. *rugosa* explants with *Agrobacteriun rhizogenes* R1000 (optical density at 600 nm, OD_600_ = 0.6), followed by co-cultivation for 48 h. After removing *A. rhizogenes* from the surface of the explants by washing ten times with sterilized water, the plant parts were placed on half-strength Murashige and Skoog (MS) medium containing 500 mg/L cefotaxime and incubated at 25 °C in the dark. Within 2 weeks, hairy roots emerged from the injured parts of the explants. The roots were allowed to grow to at least 10 cm in length before being aseptically cut from the mother plant explants. The cut portions were transferred to basal medium containing 500 mg/L cefotaxime. After a 1-month culture, the hairy roots were transferred to basal medium containing 250 mg/L cefotaxime. After a further 1-month incubation, the hairy roots were incubated on basal medium without cefotaxime to confirm elimination of *Agrobacterium*. To obtain sufficient biomass to assess the effect of different carbon sources, hairy roots were cultured in half-strength MS liquid medium. Thereafter, hairy roots [4 g (fresh weight)] were cultured in the dark in 30 mL of half-strength MS containing 100 mM different carbohydrates (sorbitol, mannitol, glucose, maltose, galactose, mannose, or sucrose) at 25 °C on a gyratory shaker operating at 100 rpm. Each carbohydrate was tested using triplicate cultures. After 5 weeks, *A*. *rugosa* hairy roots were harvested and freeze-dried to measure dry weight and perform further analyses.

### 4.2. High-Performance Liquid Chromatography and Determination of Total Phenolic Content

Rosmarinic acid was analyzed as previously described [46,47]. Dried powder (100 mg) of *A*. *rugosa* hairy roots cultured in different carbohydrates was extracted with 2 mL of 80% methanol in a sonicator for 1 h and then centrifuged at 3000× *g* for 15 min. The supernatant was then syringe-filtered into a vial and analyzed by NS-4000 HPLC system (Futecs Co., Daejeon, Republic of Korea) as previously described [29,30]. Rosmarinic acid in *A*. *rugosa* hairy roots was compared with the retention time of the standard (100 ppm rosmarinic acid) and spike test. Quantification of rosmarinic acid was performed using a calibration curve established using dilutions of the standard. Extracts were also used for total phenolic content analysis using the Folin–Ciocalteu method. Briefly, 0.1 mL of each extract from hairy root cultured in the different carbon sources was added to a tube containing 3 mL of HPLC grade water, followed by 0.5 mL of 2 N Folin and Ciocalteu phenol reagent. Each mixture was incubated for 3 min, followed by the addition of 2 mL of sodium carbonate (20%, *w*/*v*). After incubation for 3 min, the absorbance of each sample was measured at 760 nm using a spectrophotometer. Calibration curve equivalent was determined using the following gallic acid standard curve equation: y = 0.002x − 0.0226 (r^2^ = 0.9997).

### 4.3. PCR Analysis

Genomic DNA of *A*. *rugosa* hairy roots cultured in different carbohydrates was isolated using a previously described one-step DNA extraction protocol [48]. The *rol* genes (A, B, C, and D) were amplified using the primers listed in Table S1. PCR amplification was performed by 30 cycles of denaturation at 95 °C for 30 s, annealing at 57 °C for 30 s, extension at 72 °C for 1 min, followed by a final elongation step at 72 °C for 10 min. One percent agarose gel electrophoresis was used to verify the expected lengths of the *rol* A (233 bp), B (483 bp), C (464 bp), and D (525 bp).

### 4.4. Screening for Antibacterial Activity

Antimicrobial screening of *A*. *rugosa* hairy roots cultured with sucrose and fructose was performed using an established disk diffusion method [49]. *A. rugosa* hairy root powder (300 mg) was soaked in 30 mL of methanol, and the mixture was sonicated for 24 h. Afterwards, the crude extracts were filtered through filter paper. The solvent was evaporated using a rotary vacuum evaporator. The final extracts were stored at 4 °C until required for analysis. *Pseudomonas aeruginosa* (KBN-10-p01827), *P*. *aeruginosa* (KBN-10-p01828), *P*. *aeruginosa* (0225), *P*. *aeruginosa* (0254), *P*. *aeruginosa* (0826), *P*. *aeruginosa* (1113), *P*. *aeruginosa* (1378), *P*. *aeruginosa* (1731), *P*. *aeruginosa* (KCCM 11803), *Escherichia coli* (KCTC 1682), *Staphylococcus aureus* (KCTC 3881), *Salmonella paratyphi* C (KCCM 41577), *Proteus mirabilis* (KCTC 2510), *Klebsiella pneumoniae* subsp. *pneumoniae* (KCTC 2690), *Shigella flexneri* (KCTC 2517), *Chryseobacterium gleum* (KCTC 2094), and *Bacillus cereus* (KCTC 3624) were incubated at 30 °C to OD_600_ = 1.0 in nutrient broth (NB). *S. epidermidis* (KCTC 3958), *P. vulgaris* (KCTC 2512), and *Enterococcus avium* (ATCC 14025) were incubated at 100 rpm at 37 °C to OD_600_ = 1.0, in Trypticase Soy broth. Additionally, *Corynebacterium xerosis* (KCTC 3435), *Micrococcus luteus* (KCTC 3063), *Vibrio parahaemolyticus* (KCTC 2471), and *Streptococcus mutans* (KCTC 3065) were incubated at 100 rpm and 30 °C to OD_600_ = 1.0, in no. 2 enriched NB, marine agar, and Brain Heart Infusion broth, respectively. *Sphingomonas paucimobilis* (KCTC 2834) was cultured at 100 rpm at 30 °C to OD_600_ = 1.0 in Reasoner’s 2A broth (R2A). *Candida albicans* (ATCC 28367) was cultured at 100 rpm at 25 °C to OD_600_ = 1.0 in a Yeast Malt broth. Subsequently, the warm agar medium containing one hundred microliter aliquots of each culture at an OD_600_ of 1.0 was poured into a plastic plates. Four sterilized paper discs were placed on the agar plates. Each was saturated with 20 μL of *A*. *rugosa* hairy roots incubated with 0.125, 0.25, 0.5, or 1 mg/mL of sucrose or fructose. Each plate was incubated at the appropriate temperature for the particular bacterium for 24 h. Diameters of the resulting zones of growth inhibition were measured.

### 4.5. Statistical Analysis

Duncan’s multiple range test and *t*-test were performed for data from dry weight evaluation, rosmarinic acid analysis, total phenolic content analysis, and screening for antibacterial activity using SAS software (version 9.4, 2013; SAS Institute, Inc., Cary, NC, USA).

## 5. Conclusions

This study aimed to optimize carbon sources for biomass productivity and rosmarinic acid production in hairy root cultures of *A*. *rugosa* infected with *A*. *rhizogenes* R1000. Furthermore, the antimicrobial activities of *A*. *rugosa* hairy root extracts were investigated to provide insights into the synergistic antimicrobial activities of phenolic compounds derived from its extracts. According to Table 1, Table 2 and Table 3, monosaccharides (galactose and glucose) and disaccharides (sucrose) are appropriate sugar sources for hairy root growth. Sucrose is considered the best carbohydrate for phenolic compounds. Furthermore, *A*. *rugosa* hairy roots had significant antibacterial properties against *B*. *cereus* and *M*. *luteus*. In particular, *A*. *rugosa* hairy roots cultured in the presence of sucrose contained the highest levels of rosmarinic acid and total phenolics, and displayed superior antibacterial activities compared to those of *A*. *rugosa* hairy roots cultured in the presence of fructose, which contained the lowest levels of phenolic compounds. These results suggest that *A*. *rugosa* hairy root can be a good biomaterial based on its rosmarinic acid production and antibacterial effect, and that the best carbohydrate source is sucrose for *A*. *rugosa* hairy root cultures.

## Figures and Tables

**Table 1:** Effects of different carbohydrates on growth in hairy root cultures *A. rugosa*.

Carbohydrates	Dry Weight (mg/30 mL)
Fructose	0.402 ± 0.012 b ^1^
Mannitol	0.196 ± 0.015 d
Sorbitol	0.189 ± 0.023 d
Glucose	0.437 ± 0.032 ab
Maltose	0.306 ± 0.021 c
Galactose	0.462 ± 0.028 a
Mannose	0.193 ± 0.016 d
Sucrose	0.460 ± 0.034 a

^1^ Means with the same letter are not significantly different at *p* < 0.05 using DMRT.

**Table 2:** Effects of different carbohydrates on the accumulation of rosmarinic acid in hairy root cultures of *A. rugosa*.

Carbohydrates	Rosmarinic Acid (mg/g Dry Weight)
Fructose	2.069 ± 0.081 d ^1^
Mannitol	4.551 ± 0.529 c
Sorbitol	4.474 ± 0.396 c
Glucose	5.538 ± 1.234b c
Maltose	4.682 ± 1.144 c
Galactose	6.061 ± 0.359 b
Mannose	5.448 ± 0.810 bc
Sucrose	7.656 ± 0.407 a

^1^ Means with the same letter are not significantly different at *p* < 0.05 using DMRT.

**Table 3:** Effects of different carbohydrates on the total phenolic contents in hairy root cultures of *A. rugosa*.

Carbohydrates	Total Phenolic Contents (mg/g GAE)
Fructose	4.286 ± 0.404 e ^1^
Mannitol	8.738 ± 0.168 c
Sorbitol	6.619 ± 0.539 d
Glucose	8.810 ± 0.471 c
Maltose	7.643 ± 1.717 cd
Galactose	10.095 ± 0.539 b
Mannose	8.238 ± 0.606 c
Sucrose	12.714 ± 0.202 a

^1^ Means with the same letter are not significantly different at *p* < 0.05 using DMRT.

**Table 4:** Antibacterial activity of methanol extracts of *A*. *rugosa* hairy root cultured with sucrose and fructose.

Group	Bacterial Strains	Zone of Inhibition (mm)	
		Extracts from Hairy Roots Cultured with Sucrose	Extracts from Hairy Roots Cultured with Fructose
Multidrug-resistant bacteria	*P*. *aeruginosa* (KBN-10-p01827)	– ^1^	–
	*P*. *aeruginosa* (KBN-10-p01828)	–	–
	*P*. *aeruginosa* (0225)	–	–
	*P*. *aeruginosa* (0254)	–	–
	*P*. *aeruginosa* (0826)	–	–
	*P*. *aeruginosa* (1113)	–	–
	*P*. *aeruginosa* (1378)	–	–
	*P*. *aeruginosa* (1731)	–	–
Pathogens	*P*. *aeruginosa* (KCCM 11803)	–	–
	*Escherichia coli* (KCTC 1682)	–	–
	*Staphylococcus aureus* (KCTC 3881)	–	–
	*Salmonella paratyphi* C (KCCM 41577)	–	–
	*Shigella flexneri* (KCTC 2517)	–	–
	*Chryseobacterium gleum* (KCTC 2094)	–	–
	*Staphylococcus epidermidis* (KCTC 3958)	–	–
	*Streptococcus mutans* (KCTC 3065)	–	–
	*Proteus vulgaris* (KCTC 2512)	–	–
	*Proteus mirabilis* (KCTC 2510)	–	–
	*Enterococcus avium* (ATCC 14025)	–	–
	*Vibrio parahaemolyticus* (KCTC 2471)	–	–
	*Corynebacterium xerosis* (KCTC 3435)	–	–
	*Klebsiella pneumoniae* subsp. *pneumonia* (KCTC 2690)	–	–
	*Sphingomonas paucimobilis* (KCTC 2834)	–	–
	*Micrococcus luteus* (KCTC 3063)	10.7 ± 0.52 **^,2^	6.1 ± 0.34
	*Bacillus cereus* (KCTC 3624)	7.1 ± 0.21 *	6.5 ± 0.14
Pathogenic yeast	*Candida albicans* (ATCC 28367)	–	–

^1^ –, negative; ^2^ asterisks indicate significant differences (*t*-test, * *p* < 0.05, ** *p* < 0.01).

## Data Availability

Not applicable.

## Supplementary Materials

The following supporting information can be downloaded at: https://www.mdpi.com/article/10.3390/plants12040797/s1, Table S1: Gene-specific primers used to confirm the establishment of transformation.
