# Supplementary material for: Effects of Carbohydrates on Rosmarinic Acid Production and In Vitro Antimicrobial Activities in Hairy Root Cultures of Agastache rugosa
Source: Plants (Basel). 2023 Feb 10;12(4):797. doi: 10.3390/plants12040797 (PMC9959714; doi:10.3390/plants12040797)
Supplement: Supplementary file 1 [file plants-12-00797-s001.zip › plants-2182934-supplementary.pdf]

Table S1. Gene specific primers used to confirm the establishment of transformation

	**Primers**
rol A (F)	TAAGCTTGTTAGGCGTGCAA
rol A (R)	AATCCCGTAGGTTTGTTTCG
rol B (F)	TTCCTTCCACGATTTCAACC
rol B (R)	GAAAATGGCGATGAAGCATT
rol C (F)	ATGTGACAAGCAGCGATGAG
rol C (R)	CCTCACCAACTCACCAGGTT
rol D (F)	GGCACCAAAGACATCCACTT
rol D (R)	CGCAGATAGGACATGCTCAA
